# Inhibition of stromal CXCR4 impairs development of lung metastases

**DOI:** 10.1007/s00262-012-1223-7

**Published:** 2012-03-08

**Authors:** Crescenzo D’Alterio, Antonio Barbieri, Luigi Portella, Giuseppe Palma, Marianeve Polimeno, Anna Riccio, Caterina Ieranò, Renato Franco, Giosuè Scognamiglio, Jane Bryce, Antonio Luciano, Domenica Rea, Claudio Arra, Stefania Scala

**Affiliations:** 1 Department of Oncological Immunology, National Cancer Institute “G. Pascale”, Via Semmola., 80131 Naples, Italy; 2 Animal Facility, National Cancer Institute “G. Pascale”, Naples, Italy; 3 Department of Pathology, National Cancer Institute “G. Pascale”, Naples, Italy; 4 Clinical Trials Unit, National Cancer Institute “G. Pascale”, Naples, Italy

**Keywords:** Pulmonary metastases, CXCR4, Heterozygote mice, Plerixafor, Melanoma

## Abstract

Compelling evidence has emerged in recent years indicating that stromal cells play a critical role in disease progression. CXCR4 is a G-protein-coupled receptor with a major role in lymphocyte homing. Its ligand, CXCL12, is a highly efficient chemotactic factor for T cells, monocytes, pre-B cells, dendritic cells and myeloid bone marrow-derived cells (BMDCs). In addition, the CXCR4–CXCL12 axis plays a central role in tumor growth and metastasis. To evaluate the effect of genetic CXCR4 reduction on metastasis development, murine melanoma B16 cells were injected into the tail vein of C57BL/6 CXCR4^+/+^ and CXCR4^+/−^ mice in the presence of the CXCR4 inhibitor, Plerixafor (previously named AMD3100). Although lung metastases developed in wild-type CXCR4^+/+^ and heterozygote CXCR4^+/−^ mice, nodules were significantly smaller in the latter. CXCR4 pharmacological inhibition by Plerixafor further reduced lung metastases in CXCR4^+/−^ mice, preserving the pulmonary architecture (4.18 ± 1.38 mm^2^ vs. 1.11 ± 0.60 mm^2^, *p* = 0.038). A reduction in LY6G-positive myeloid/granulocytic cells and in p38 MAPK activation was detected in lungs from CXCR4^+/−^ mice compared to CXCR4^+/+^ mice [LY6G-positive myeloid CXCR4^+/−^ vs. CXCR4^+/+^ (*p* = 0.0004); CXCR4^+/+^ vs. CXCR4^+/+^ Plerixafor-treated (*p* = 0.0031)] suggesting that CXCR4 reduction on myeloid-derived cells reduced their recruitment to the lung, consequently impairing lung metastases. Our findings argue in favor of a specific role of CXCR4 expressed in stromal cells that condition the pro-tumor microenvironment. In this scenario, CXCR4 antagonists will target neoplastic cells as well as the pro-tumor stromal microenvironment.

## Introduction

The microenvironment of a developing tumor is composed of proliferating malignant cells, tumor stroma, blood vessels, and infiltrating inflammatory cells [1]. It is a unique environment created and dominated by tumor cells that establish specific interactions with the neighboring cells to promote tumor progression and metastasis [1]. Compelling evidence has emerged in recent years indicating that ‘stromal cells’ play a critical role in disease progression. The interactions of cancer cells with components of their tumor microenvironment are bidirectional and crucial for cancer progression [2–4]. Tumor cells communicate with their surrounding microenvironment via a network of secreted growth factors, cytokines, and chemokines [5, 6]. Among the chemokine receptors, CXCR4 is the most involved in cancer, as it is expressed in at least 23 different types of cancers [6]. The axis CXCR4–CXCL12 plays an important role in tumorigenicity, proliferation, metastasis, and angiogenesis in cancer [7–11], and CXCR4 expression affects prognosis in melanoma [12], colon cancer [13], lung cancer [14], renal cell carcinoma, and [15, 16] osteosarcoma [17–19]. In normal adult tissues, CXCR4 and CXCL12 are constitutively expressed in a wide range of tissue [20–22], and mice lacking CXCR4 die in utero or shortly after birth displaying defects in the hematopoietic and nervous systems [23]. Identical defects are observed in mice lacking CXCL12, suggesting a monogamous relationship between CXCR4 and CXCL12 [24–26]. Nevertheless, recent evidence has shown that CXCL12 also binds the recently deorphanized chemokine receptor CXCR7 [27, 28]. In addition, the axis CXCR4–CXCL12 regulates reactive infiltrates into tumors, MSCs (mesenchymal stromal cells) [29, 30], monocyte/macrophage lineage cells, and T lymphocytes [31]. MSCs constitutively secrete the chemokine CXCL12 (SDF-1), which in turn promotes tumor progression by recruitment of endothelial progenitor cells into tumors [32]. Increasing evidence supports a critical role for the interaction between cancer cells and myeloid bone marrow-derived cells (BMDCs) during tumor growth and metastasis [33]; These cells are mobilized into blood circulation and infiltrate the neoplastic tissues from early stages of tumor growth in response to tumor- and stroma-derived cytokines [1, 32–34]. It has been well established that tumor-associated macrophages (TAMs) and myeloid differentiation antigen (Gr-1)-positive myeloid BMDCs can promote angiogenesis and tumor progression [34]. Gr-1-positive myeloid BMDCs express CXCR4 and VEGFR1, the former being essential for recruitment of myeloid differentiation antigen (Gr-1)-positive BMDCs [35]. Indeed, VEGFR1 and CXCR4 independently exerted a promigratory effect in myeloid BMDCs by activating p38 mitogen-activating protein kinase [35–37]. To evaluate the effect of genetic CXCR4 reduction on lung metastases, murine melanoma B16 cells were inoculated in a model of murine C57Bl/6 mice CXCR4^+/+^ and CXCR4^+/−^.

## Materials and methods

### Cell culture

A B16 murine melanoma cell line, syngenic for C57Bl/6 mice was morphologically authenticated and maintained in IMDM, 10% FBS, 1% P&S and 1% L-Glutammine at 37°C with 5% CO_2_. The CCRF-CEM cell lines were from ATCC and cultured under conditions provided by the manufacturer. ATCC Molecular Authentication Resource Center provides a variety of applications to identify and characterizing the cell lines, including cloning and gene synthesis, real-time PCR analyses, site-directed mutagenesis, sequencing, STR, SNP, and fingerprint analyses.

### Real time PCR

Total RNA from dissected fresh tissues from CXCR4^+/+^ and CXCR4^+/−^ mice were extracted using RNA*later* RNA Stabilization Reagent (Qiagen, Hilden) to immediately stabilizes RNA in tissue samples (so as to preserve the gene expression profile) and RNeasy Mini Kit quick spin columns (Qiagen), according to the manufacturer’s instructions. DNase-treated RNA (200 ng) was reverse transcribed by Superscript II RNase H-reverse transcriptase according to the manufacturer’s instructions (Invitrogen-Life Technologies, Carlsbad, CA, USA). Real time-PCR was carried out using about 10 ng of cDNA in 25 μl final of SYBR Green reaction mixture. An ABI Prism 7000 (Applied Biosystems) robocycler was used for the amplification. For both CXCR4 and CXCL12, cycling conditions of the PCR were as follows: initial denaturation (10 min at 95°C) followed by 40 cycles of denaturation (15 s at 95°C), annealing (30 s at 60°C) synthesis (1 min at 72°C), followed by final extension (7 min at 72°C). The gene-specific mouse primers used for the amplification were as follows: CXCR4: 5′-ACCTCTACAGCAGCGTTCTCA-3′ (forward); 5′-GGTGGCGTGGACAATAG-3′ (reverse); CXCL12: 5′-GCCCTGCTCTGTCTGCTAAA-3′ (forward); 5′-CCTGGCCTTCATGGGATTGT-3′ (reverse); GAPDH: 5′-TGGCCTTCCGTGTTCCTACCC-3′(forward)5′-TCTCCAGGCGGCACGTC-3′ (reverse). Subsequently, CXCR4 and CXCL12 mRNA was quantified comparing its expression to GAPDH mRNA levels. Samples were run in triplicate.

### Immunoblotting analysis

Total protein was extracted from dissected mice tissues and from B16 melanoma cells, after homogenization in lysis buffer (40 mM Hepes pH 7.5, 120 mM NaCl, 5 mM MgCl_2_, 1 mM EGTA, 0.5 mM EDTA, 1% Triton X-100) containing protease (Complete Tablets EDTA-free; Roche) and phosphatase (20 mM a-glycerol-phosphate, 2,5 mM Na-pyrophosphate) inhibitors. CCRF-CEM cell lines were used as CXCR4 positive control. The following primary antibodies were used: anti-CXCR4 (Abcam; ab2074, 1:1,000 diluition), anti-CXCL12 (R&D Systems; mab350, 1:500 diluition;); anti phospho-p38 MAPK and anti p38 MAPK, 1:1,000 diluition (Cell Signaling Technology; code 4511 and code 9212, respectively). The alpha-tubulin (Santa Cruz Biotech; clone B-7: sc-5286 1:3,000 diluition) used as housekeeping controls. Appropriate Anti IgG coupled with peroxidase were used as secondary antibodies (Santa Cruz Biotech, Santa Cruz, CA, USA) and the signal was revealed through Chemoluminescent detection kit (ECL detection kit; Amersham Biosciences, Freiburg, Germany). Optical density of bands was quantified using the ImageJ software.

### Cell migration assay

Migration was assayed in 6-well Transwell chambers of Corning 8-μm pore filter (Corning, NY, USA). We placed 6 × 10^5^ B16 cells in IMDM containing 0.5% BSA (migration media) on the upper chamber filter that was precoated with collagen (human collagen type I/III) and fibronectin (10 g/ml each). Medium supplemented with recombinant human CXCL12 (used at 100 ng/ml each) (R&D Systems; NS-350) with and without Plerixafor (used at 5 μM each) was placed in the lower chamber. After 16 h incubation, the number of invading cells were counted in ten different fields (HPF ×400 magnification).

### Animal experiments

Ten female C57BL/6 homozygote CXCR4^+/+^ mice (8–10 weeks old) weighing approximately 18–20 g were purchased from Harlan Laboratory (Bar Harbor, ME, USA) and ten female C57BL/6 heterozygote CXCR4^+/−^ mice (8–10 weeks old) kindly provided by Prof. De Felice, Biogem IRGS (Ariano Irpino, Italy). The research protocol was approved, and mice were housed three to five per cage with food and water available ad libitum and maintained on a 12-h light/dark cycle under standard and specific pathogen-free conditions in the Animal Care Facility of National Cancer Institute “G. Pascale” in accordance with the institutional guidelines of the Italian Ministry of Health Animal Care and Use Committee. Mice were acclimatized for 1 week before being injected with cancer cells.

### In vivo metastasis assays

B16 murine melanoma cells in exponential growth phase were harvested and washed twice in PBS before injection. Cell viability was >95% as determined by trypan blue dye exclusion. Mice were injected into the tail veins with 5 × 10^5^ B16 cells suspended in 200 μl phosphate-buffered saline (PBS). Five mice per group were inoculated with (1) B16 cells and (2) B16 cells pretreated with 10 μg/ml Plerixafor for 30 min and, after inoculation, mice were treated twice a day with 1.25 mg/kg Plerixafor (Sigma Life Science) for 2 weeks, 5 days for week. Mice were euthanized 19 days after the tumor cells injection for gross inspection of organs and subsequent analysis.

### Immunohistochemical analysis

Mice tissues (lungs, liver, lymph nodes, spleen) were fixed in 10% buffered formalin, paraffin-embedded and subsequently sectioned into 3-μm slices. The sections were stained with haematoxylin/eosin to evaluate metastasis (R.F. and C.D.). Histological evidence of metastases were measured and summed using a computer-assisted image measurement program by a microscope (BX51 microscope and DP-1 microsope digital camera; Olympus Japan). Monocyte/granulocyte infiltration was evaluated through IHC on lung section. Staining was conducted using myeloid differentiation antigen LY6G. (Rat Anti-Mouse Ly-6G, clone 1A8, code No. 551459; BD Pharmingen) diluited 1:1,000. Monocyte/granulocyte count was performed blindly by two operators (R.F. and C.D.) with a magnification of ×200 field (×20 objective and ×10 ocular), on three areas with high density of macrophages/monocytes, selected at low power magnification.

### Statistical analysis

The values given are means ± standard deviation. The significance of difference between the experimental groups and controls was assessed by unpaired Student’s *t* test, using Instat Software (GraphPad, San Diego, CA, USA).

## Results

### CXCR4 and CXCL12 expression in C57BL/6 CXCR4^+/−^ mice

**Fig. 1:**
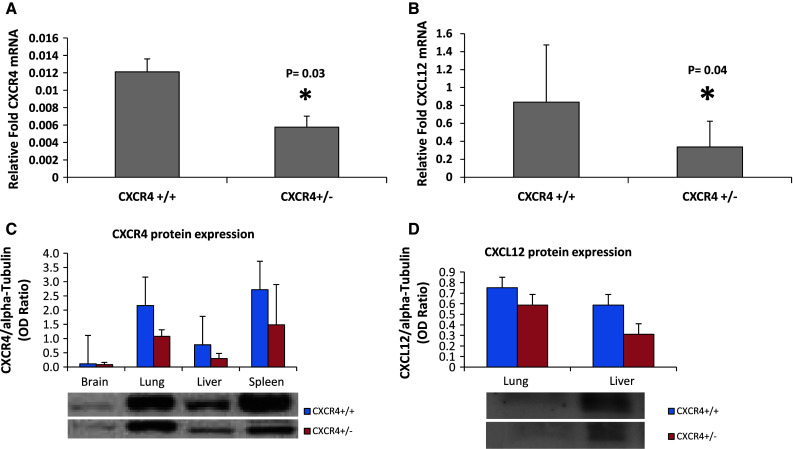
CXCR4 and CXCL12 expression in C57BL/6 CXCR4^+/+^ and CXCR4^+/−^. Quantitative RT-PCR analysis for CXCR4 (**a**) and CXCL12 (**b**) gene expression on normal lung tissue. Immunoblotting for CXCR4 (**c**), CXCL12 (**d**) in normal tissue from homozygote (CXCR4^+/+^) and heterozygote mice (CXCR4^+/−^)

**Fig. 2:**
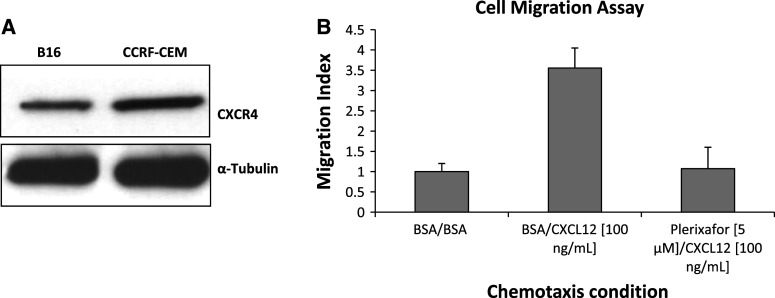
B16 cells express functional CXCR4. Immunoblot for CXCR4 in B16 cells (**a**); B16 migration toward CXCL12, inhibited by Plerixafor (**b**)

To evaluate the role of genetic reduction of CXCR4 in lung metastases development, CXCR4^+/−^ mice were utilized, since CXCR4^−/−^ mice are embryonic ally lethal. Level of CXCR4 and CXCL12 transcripts in CXCR4^+/+^ and CXCR4^+/−^ C57BL/6 mice were evaluated in normal lung tissue. CXCR4^+/−^ mice lung expressed lower CXCR4 and CXCL12 mRNA compared to wild-type mice (Fig. 1a, b). Reduction of CXCR4 and CXCL12 protein expression in CXCR4^+/−^ C57BL/6 mice was also demonstrated through immunoblotting analysis (Fig. 1c, d). Additionally, it was shown that murine B16 melanoma cells express functional CXCR4 (Fig. 2a, b, respectively).

### Inhibition of CXCR4-CXCL12 axis reduced lung metastases development

**Fig. 3:**
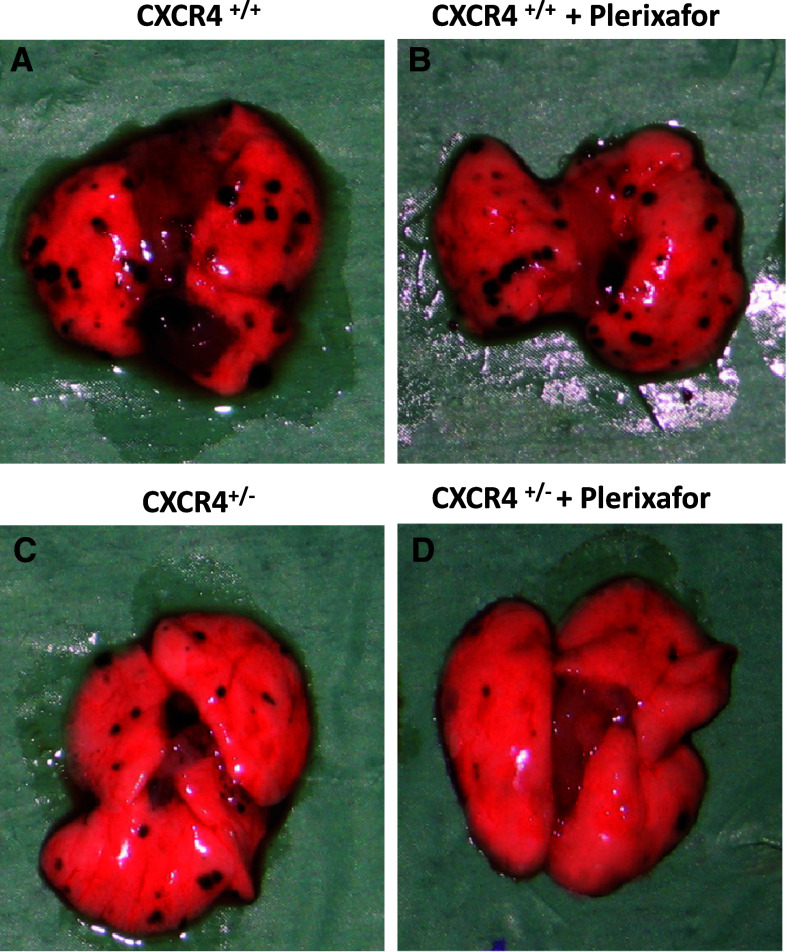
Reduction in lung metastases development in CXCR4^+/−^mice. Gross inspection of representative lungs: CXCR4^+/+^ and Plerixafor-treated CXCR4^+/+^ (**a**, **b**) and CXCR4^+/−^and CXCR4^+/−^ Plerixafor-treated mice (**c**, **d**)

**Fig. 4:**
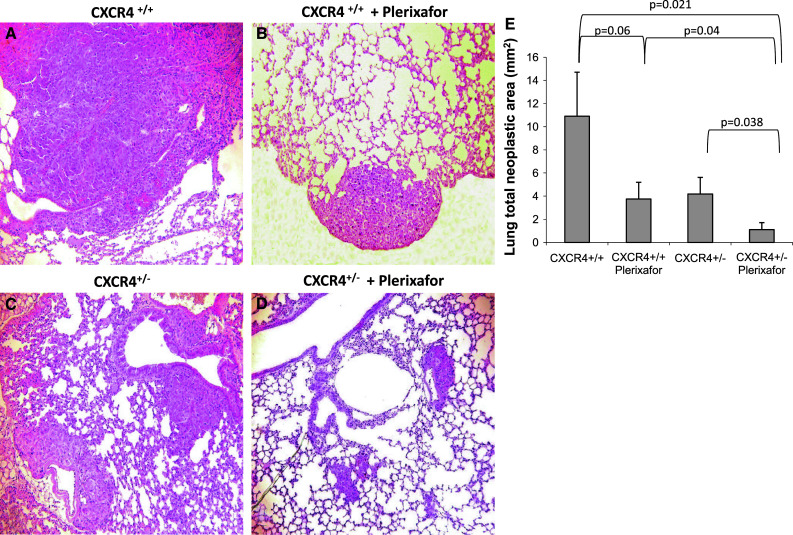
Microscopic evaluation of lung metastases. Microscopic evaluation of representative lungs. H&E in CXCR4^+/+^ and Plerixafor-treated CXCR4^+/+^ (**a**, **b**) and CXCR4^+/−^and CXCR4^+/−^ Plerixafor-treated mice (**c**, **d**); (**e**) Lung total neoplastic area expressed in mm^2^

Murine melanoma B16 cells (5 × 10^5^) were injected into CXCR4^+/+^ and CXCR4^+/−^ C57BL/6 and mice were treated for 10 days with 1.25 mg/kg of Plerixafor. At day 19, mice were sacrificed and organs were examined. In Fig. 3, representative lungs are shown; reduced metastases were detected in Plerixafor-treated mice on both CXCR4^+/+^ and CXCR4^+/−^ mice lung. Nevertheless, CXCR4^+/−^ mice lung showed less metastases compared to wild-type (Fig. 3). In Fig. 4, lung tissue microscopic evaluation confirmed the massive substitution of lung tissue with neoplastic tissue in CXCR4^+/+^ mice (10.90 ± 3.81 mm^2^); in the Plerixafor-treated CXCR4^+/+^ mice, the metastatic nodules were reduced (3.58 ± 1.38 mm^2^). Although the number of lung metastases was comparable in CXCR4^+/−^ and CXCR4^+/+^ lungs, in CXCR4^+/−^ lungs, the nodules were smaller and a further reduction was detected in CXCR4^+/−^ Plerixafor-treated mice. Reduction in total neoplastic area was reported in Plerixafor-treated CXCR4^+/+^ and CXCR4^+/−^ mice compared to untreated mice, and in CXCR4^+/−^ Plerixafor compared to untreated CXCR4^+/−^ (4.18 ± 1.38 mm^2^ vs. 1.11 ± 0.60 mm^2^, *p* = 0.038) and to CXCR4^+/+^ (1.11 ± 0.60 mm^2^ vs. 10.90 ± 3.81 mm^2^, *p* = 0,021; Fig. 4).

### LY6G-positive myeloid/granulocytic cells and Phospho-p38 decrease in CXCR4^+/−^ tissues

**Fig. 5:**
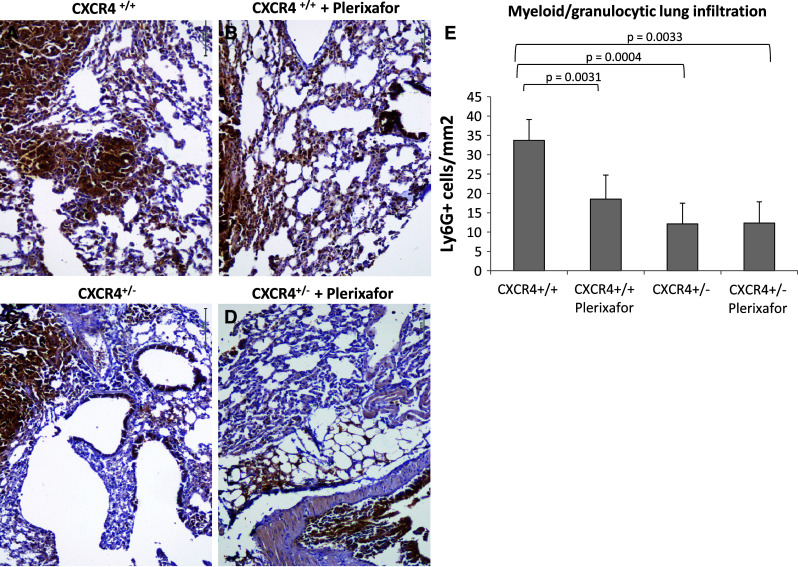
Decrease of myeloid differentiation antigen LY6G^+^ cells in CXCR4^+/−^ lungs. LY6G^+^ myeloid recruitment in peritumoral lung tissue from CXCR4^+/+^ and CXCR4^+/+^ Plerixafor-treated (**a**, **b**) and from CXCR4^+/−^and CXCR4^+/−^ Plerixafor-treated (**c**, **d**) (×200 magnification); (**e**) LY6G positive cells number in CXCR4^+/+^ and C57BL/6 CXCR4^+/−^ mice in the presence or absence of Plerixafor treatment

**Fig. 6:**
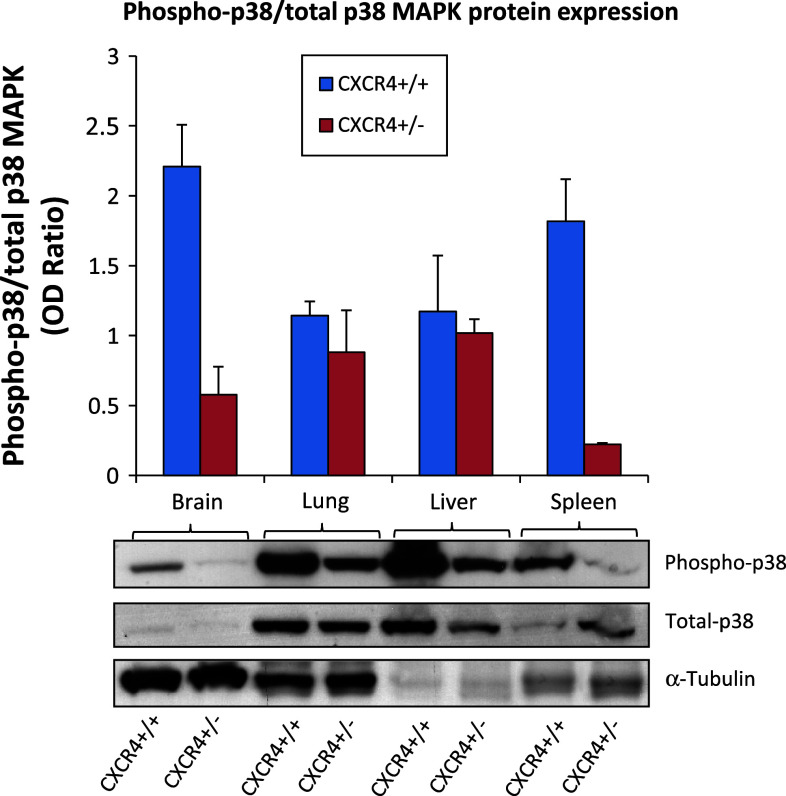
Decrease in Phopho-p38 MAPK in CXCR4^+/−^ tissue. Immunoblotting for P38 MAPK, Phopho-p38 MAPK signals decrease in CXCR4^+/−^ compared to CXCR4^+/+^ normal tissue; (mean ± SD of ratio in phospho-p38/Total p-38 tubulin normalized). Brain 2.2 versus 0.5; lung 1.1 versus 0.9; liver 1.2 versus 1; spleen 1.8 versus 0.2 for CXCR4^+/+^ versus CXCR4^+/−^, respectively

The above results indicate that genetic reduction of CXCR4 in C57BL/6 mice reduced B16 lung metastase development. Considering that the inoculated cell line B16 expresses functional CXCR4 in both mice models, the results relied on a lower CXCR4 expression in the multiplicity of cells that form the tumor stroma. Recent evidence suggests that CXCR4 expressing myeloid bone marrow-derived cells (BMDCs) play a critical role in lung metastasis [35, 38, 39]. Although not able to define the bone marrow origin, evaluation of LY6G-positive myeloid/granulocytic cells was conducted in lung section through IHC. As demonstrated in Fig. 5, a significant decrease in LY6G-positive myeloid cells was detected in lung from CXCR4^+/−^ mice compared to CXCR4^+/+^ mice [LY6G-positive myeloid CXCR4^+/−^ vs. CXCR4^+/+^ (*p* = 0.0004); CXCR4^+/+^ vs. CXCR4^+/+^ Plerixafor-treated (*p* = 0.0031)]. Since CXCR4 signal transduction in myeloid BMDCs rapidly increased p38 MAPK phosphorylation, phospho-p38 MAPKinase level was evaluated in tissues from CXCR4^+/+^ and CXCR4^+/−^ mice. As shown in Fig. 6, the level of phospho-p38 MAPK decreased in the CXCR4^+/−^-derived tissues.

## Discussion

Metastasis occurs in an organ-specific and highly organized manner. Tumors metastasize to preferred sites by diverse determinants [40], and increasing evidence has shown that the microenvironment can modulate metastatic potential [41]. This article has focused on the role of genetic CXCR4 in the development of lung metastases. Murine B16 cells were injected in C57BL/6 CXCR4^+/+^ and CXCR4^+/−^ mice in the presence of the CXCR4 inhibitor, Plerixafor. Although lungs metastases were detected in both CXCR4^+/+^ and CXCR4^+/−^ mice, tumor burden was significantly less in CXCR4^+/−^ mice, Plerixafor treatment further reduced the size and number of lung metastases more effectively in CXCR4^+/−^ mice, preserving the pulmonary architecture. As expected, CXCR4 expression was reduced in the CXCR4^+/−^ mice; concomitantly the ligand CXCL12 was also reduced. This result, although previously reported [42], furnished a possible mechanism to the reduced lung metastases development. Constitutive secretion of CXCL12 by the stromal cells induces migration and adhesion of neoplastic cells to the stromal cells via CXCR4 activation. In solid tumors, different stromal cells express CXCL12 and/or its receptors creating paracrine interactions that promote tumor progression. Moreover, CXCL12 is significantly involved in recruitment of various bone marrow–derived cells (BMDC) expressing CXCR4. These cells are reduced in CXCR4^+/−^ mice lung, and a reduction in the activity of p38 MAPK activity, a CXCR4-downstream target in BMDC, was also registered in CXCR4^+/−^ mice lung [35]. Since activation of the CXCL12 pathway may promote cancer cell survival, invasion, and stem and/or tumor-initiating cell phenotype, blocking the CXCR4-CXCL12 pathway may be a valid strategy to target various components in solid tumors**.**

Multiple preclinical studies have converged on the finding that anti-CXCL12 agents can significantly delay primary tumor growth and metastasis when treatment is started at or close to the time of tumor implantation. However, previous evidence has shown that blockade of the CXCL12 pathway had minor antitumor effects on established tumors. One potential setting in which blockade of the CXCL12 pathway may be more widely efficacious is in preventing or delaying metastasis. Focused settings, such as relapsed glioblastoma, neoadjuvant colorectal and breast cancer, and advanced prostate cancer would be ideal for evaluating CXCR4-inhibiting agents in the development and progression of metastases.

The first and potent CXCR4 antagonist, Plerixafor, is Food and Drug Administration (FDA) approved for Stem Cell Mobilization in patients with Multiple Myeloma and Non-Hodgkin lymphoma. With the aim of inhibiting the metastatic diffusion, several other anti CXCR4 inhibitors are in clinical development such as peptidic inhibitors (BK-T140 and CTCE-9908), antibodies (MDX-1338), and small molecules (POL6326).

Several Phase I studies are ongoing in which newly developed anti-CXCR4 antagonists (POL6326, BKT140, TG0054, NOX-A12) are challenged in hematological neoplasias as mobilizing agents, but there are no ongoing clinical trials in solid tumors. To assess a possible role for stromal CXCR4 in metastatic dissemination, an heterozygote mice model for CXCR4 was studied. Reduced numbers and size of lung metastases were induced in CXCR4^+/−^ mice further reduced by Plerixafor. Reduction in lung metastases in CXCR4^+/−^-derived lungs were associated with reduced myeloid CXCR4 positive-LY6G and a reduced p38 MAPK signal transduction. These results suggest that a reduced number of functional myeloid CXCR4 positive-LY6G may determine a less favorable microenvironment. Our findings argue in favor of a specific role of CXCR4 expressed in stromal cells that conditioned the pro-tumor microenvironment. In this scenario, CXCR4 antagonists will target neoplastic cells as well as the pro-tumor stromal microenvironment.

## References

[1] Mantovani A, Allavena P, Sica A, Balkwill F (2008). Cancer-related inflammation. Nature.

[2] De Visser KE, Coussens LM (2006). The inflammatory tumor microenvironment and its impact on cancer development. Contrib Microbiol.

[3] Raman D, Baugher PJ, Thu YM, Richmond A (2007). Role of chemokines in tumor growth. Cancer Lett.

[4] Ben-Baruch A (2006). The multifaceted roles of chemokines in malignancy. Cancer Metas Rev.

[5] Vicari AP, Caux C (2002). Chemokines in cancer. Cytokine Growth Factor Rev.

[6] Balkwill F (2004). Cancer and the chemokine network. Nat Rev.

[7] Ehtesham M, Mapara KY, Stevenson CB, Thompson RC (2008). CXCR4 mediates the proliferation of glioblastoma progenitor cells. Cancer Lett.

[8] Lee BC, Lee TH, Avraham S, Avraham HK (2004). Involvement of the chemokine receptor CXCR4 and its ligand stromal cell-derived factor 1alpha in breast cancer cell migration through human brain microvascular endothelial cells. Mol Cancer Res.

[9] Mori T, Doi R, Koizumi M, Toyoda E, Ito D, Kami K (2004). CXCR4 antagonist inhibits stromal cell derived factor 1-induced migration and invasion of human pancreatic cell. Mol Cancer Ther.

[10] Chen GS, Yu HS, Lan CC, Chow KC, Lin TY, Kok LF (2006). CXC chemokine receptor CXCR4 expression enhances tumorigenesis and angiogenesis of basal cell carcinoma. Br J Dermatol.

[11] Bartolomé RA, Gálvez BG, Longo N, Baleux F, Van Muijen GN, Sánchez-Mateos P (2004). Stromal cell-derived factor-1 promotes melanoma cell invasion across basement membranes involving stimulation of membrane-type 1 matrix metalloproteinase and Rho GTPase activities. Cancer Res.

[12] Scala S, Ottaiano A, Ascierto PA, Cavalli M, Simeone E, Giuliano P (2005). Expression of CXCR4 predicts poor prognosis in patients with malignant melanoma. Clin Cancer Res.

[13] Kim J, Takeuchi H, Lam ST, Turner RR, Wang HJ, Kuo C (2005). Chemokine receptor CXCR4 expression in colorectal cancer patients increases the risk for recurrence and for poor survival. J Clin Oncol.

[14] Minamiya Y, Saito H, Takahashi N, Ito M, Imai K, Ono T (2010). Expression of the chemokine receptor CXCR4 correlates with a favorable prognosis in patients with adenocarcinoma of the lung. Lung Cancer.

[15] D’Alterio C, Cindolo L, Portella L, Polimeno M, Consales C, Riccio A (2010). Differential role of CD133 and CXCR4 in renal cell carcinoma. Cell Cycle.

[16] D’Alterio C, Consales C, Polimeno M, Franco R, Cindolo L, Portella L (2010). Concomitant CXCR4 and CXCR7 expression predicts poor prognosis in renal cancer. Curr Cancer Drug Targets.

[17] Oda Y, Yamamoto H, Tamiya S, Matsuda S, Tanaka K, Yokoyama R (2006). CXCR4 and VEGF expression in the primary site and the metastatic site of human osteosarcoma: analysis within a group of patients, all of whom developed lung metastasis. Mod Pathol.

[18] Perissinotto E, Cavalloni G, Leone F, Fonsato V, Mitola S, Grignani G (2005). Involvement of chemokine receptor 4/stromal derived factor 1 system during osteosarcoma tumour progression. Clin Cancer Res.

[19] Laverdiere C, Hoang BH, Yang R, Sowers R, Qin J, Meyers PA (2005). Messenger RNA expression levels of CXCR4 correlate with metastatic behavior and outcome in patient with osteosarcoma. Clin Cancer Res.

[20] Bleul CC, Wu L, Hoxie JA, Springer TA, Mackay CR (1997). The HIV coreceptors CXCR4 and CCR5 are differentially expressed and regulated on human T lymphocytes. Proc Natl Acad Sci USA.

[21] Tashiro K, Tada H, Heilker R, Shirozu M, Nakano T, Honjo T (1993). Signal sequence trap: a cloning strategy for secreted proteins and type I membrane proteins. Science.

[22] Federsppiel B, Melhado IG, Duncan AM, Delaney A, Schappert K, Clark-Lewis I (1993). Molecular cloning of the cDNA and chromosomal localization of the gene for a putative seven-transmembrane segment (7-TMS) receptor isolated from human spleen. Genomics.

[23] Ma Q, Jones D, Borghesani PR, Segal RS, Nagasawa T, Kishimoto T (1998). Impaired B-lymphopoiesis, myelopoiesis, and derailed cerebellar neuron migration in CXCR4- and SDF-1-deficient mice. Proc Natl Acad Sci USA.

[24] Nagasawa T, Hirota S, Tachibana K, Takakura N, Nishikawa S, Kitamura Y (1996). Defects of B-cell lymphopoiesis and bone-marrow myelopoiesis in mice lacking the CXC chemokine PBSF/SDF-1. Nature.

[25] Tachibana K, Hirota S, Iizasa H, Yoshida H, Kawabata K, Kataoka Y (1998). The chemokine receptor CXCR4 is essential for vascularization of the gastrointestinal tract. Nature.

[26] Zou Y, Kottman A, Kuroda M, Taniuchi I, Littman D (1998). Function of the chemokine receptor CXCR4 in haematopoiesis and in cerebellar development. Nature.

[27] Burns JM, Summers BC, Wang Y, Melikian A, Berahovich R, Miao Z (2006). A novel chemokine receptor for SDF-1 and I-TAC involved in cell survival, cell adhesion, and tumor development. J Exp Med.

[28] Balabanian K, Lagane B, Infantino S, Chow KY, Harriague J, Moepps B (2005). The chemokine SDF-1/CXCL12 binds to and signals through the orphan receptor RDC1 in T lymphocytes. J Biol Chem.

[29] Kalluri R, Zeisberg M (2006). Fibroblasts in cancer. Nat Rev.

[30] Orimo A, Weinberg RA (2006). Stromal fibroblasts in cancer: a novel tumor-promoting cell type. Cell Cycle.

[31] Liyanage UK, Moore TT, Joo HG, Tanaka Y, Herrmann V, Doherty G (2002). Prevalence of regulatory T cells is increased in peripheral blood and tumor microenvironment of patients with pancreas or breast adenocarcinoma. J Immunol.

[32] Orimo A, Gupta PB, Sgroi DC, Arenzana-Seisdedos F, Delaunay T, Naeem R (2005). Stromal fibroblasts present in invasive human breast carcinomas promote tumor growth and angiogenesis through elevated SDF-1/CXCL12 secretion. Cell.

[33] Bergfeld SA, DeClerck YA (2010). Bone marrow-derived mesenchymal stem cells and the tumor microenvironment. Cancer Metastat Rev.

[34] Murdoch C, Muthana M, Coffelt SB, Lewis CE (2008). The role of myeloid cells in the promotion of tumour angiogenesis. Nat Rev Cancer.

[35] Hiratsuka S, Duda DG, Huang Y, Goel S, Sugiyama T, Nagasawa T, Fukumura D, Jain RK (2011). C-X-C receptor type 4 promotes metastasis by activating p38 mitogen-activated protein kinase in myeloid differentiation antigen (Gr-1)-positive cells. Proc Natl Acad Sci USA.

[36] Dawson MR, Duda DG, Fukumura D, Jain RK (2009) VEGFR1-activity-independent metastasis formation. Nature 461. doi:10.1038/nature08254 10.1038/nature08254 PMC3065241 19759568

[37] Bendall LJ, Baraz R, Juarez J, Shen W, Bradstock KF (2005). Defective p38 mitogen-activated protein kinase signaling impairs chemotaxic but not proliferative responses to stromal-derived factor-1α in acute lymphoblastic leukemia. Cancer Res.

[38] Kim SY, Lee CH, Midura BV, Yeung C, Mendoza A, Hong SH (2008). Inhibition of the CXCR4/CXCL12 chemokine pathway reduces the development of murine pulmonary metastases. Clin Exp Metastasis.

[39] Burger JA, Stewart DJ, Wald O, Peled A (2011). Potential of CXCR4 antagonists for the treatment of metastatic lung cancer. Expert Rev Anticancer Ther.

[40] Nguyen DX, Bos PD, Massague J (2009). Metastasis: from dissemination to organ specific colonization. Nat Rev Cancer.

[41] Joyce JA, Pollard JW (2009). Microenvironmental regulation of metastasis. Nat Rev Cancer.

[42] Parachikova A, Cotman CW (2007). Reduced CXCL12/CXCR4 results in impaired learning and is downregulated in a mouse model of Alzheimer disease. Neurobiol Dis.

